# An Insight into Preparatory Methods and Characterization of Orodispersible Film—A Review

**DOI:** 10.3390/ph15070844

**Published:** 2022-07-09

**Authors:** Ahmad Salawi

**Affiliations:** Department of Pharmaceutics, College of Pharmacy, Jazan University, Jazan 45142, Saudi Arabia; asalawi@jazanu.edu.sa

**Keywords:** orodispersible film, formulation, solvent casting method, evaluation techniques, disintegration time

## Abstract

Over the past few decades, researchers and companies have been trying to develop novel drug delivery systems to ensure safety, efficacy, compliance, and patient acceptability. Nowadays drug discovery and development are expensive, complex, and time-consuming processes, but trends are moving toward novel drug delivery systems. This delivery system helps to achieve drug response by local and systemic action through different routes. This novel approach of preparing orodispersible films (ODFs) provides benefits to paediatric, geriatric, and bedridden patients. This review paper aims to provide details on the preparation, characterization, and evaluation of ODFs; it also aims to focus on the positive and negative factors that affect film formulation and give an insight into potential drug candidates and polymers for use in ODFs. ODFs are effective, safe, and have good bioavailability as compared to fast-disintegrating tablets. The novel approach has various advantages because it provides instant effects in emergency situations and in schizophrenic and dysphasic patients without the need for taking water, the films disintegrating within a few seconds in the oral cavity. The solvent casting method is the most frequently used technique to develop ODFs, using film-forming polymers, which have a fast disintegration time, improved drug dissolution, and better drug contents.

## 1. Introduction

The oral mucosal epithelium is a multi-cell layer (40–50 cells thick) and is made up of proteins and carbohydrates. Mucus is a gel-like fluid released from the mucosal epithelium, having 90–99% water content, the remaining components being (water-insoluble) glycoproteins, nucleic acids, electrolytes, and enzymes. However, the salivary ducts release parotid and saliva from the salivary glands near the sublingual canals, and glands secrete around 1–2 mL of saliva in sixty seconds. Water, mucus, lysozymes, amylase, immunoglobulin, clotting factors, and mineral salts are the components of saliva, and saliva acts as a barrier. The mucosal epithelium layer consists of two areas—a hydrophilic area and a lipophilic area. The oral mucosa has a higher permeability as compared to the intestine and epidermis and has around 4–4000 times more penetration power than the skin. The mucosal epithelium layer has two absorption routes for drugs—the transcellular and paracellular paths. The amphiphilic, hydrophilic, or hydrophobic nature determines the absorption capacity of drugs. Hydrophilic drug penetration is facilitated by the polar nature of the intercellular space, while lipophilic structure facilitates drug molecules with a high partition coefficient [1,2].

The oral route is the best and preferred route of drug administration in humans for the aim of obtaining relief from pain, and it also helps several drug candidates to exert various intended effects and secure patient compliance. Drugs designed to deliver their effects via the oral route are prepared using oral drug delivery systems. Drugs administered by this route are manufactured easily and lower costs are required as they do not necessitate sterile conditions [3,4]. Currently, several manufactured pharmaceutical drugs are available on the market and formulated in the form of tablets, powders, liquids, capsules, granules, and syrups. Pediatric, geriatric, and paralyzed patients have difficulty swallowing drugs that are prepared in capsule or tablet form due to swallowing problems which prevent the administration of drugs in solid dosage form [5].

To overcome these problems, a novel dosage form has been developed with the aim of providing drugs quickly and efficiently via the oral route without the use of water. This novel dosage form has been named the oral fast-dissolving drug delivery system. This approach helps to secure the compliance of patients by providing drugs at the site of action more quickly and efficiently, with prompt disintegration, dissolution, and administration, without the need for chewing and swallowing [6]. This novel approach has changed the conventional drug delivery methodology for oral drugs, and by this novel drug delivery system, drug release can be achieved with the modified dosage form, replacing oral tablets with oral films that have fast disintegration. Conventional tablets are easily broken down and require substantial packaging for handling, storage, and transportation, while oral films are flexible, easy to handle, and can be stored for longer use [7].

According to the ninth edition of the European Pharmacopoeia (Ph. Eur.), orodispersible films are formulations that are intended for the delivery of drugs by oral administration via the oral cavity. The Ph. Eur. states that ODFs mainly consist of a polymer with film-forming capacity, which serves as an active pharmaceutical ingredient or drug carrier. Plasticizers are also used to ensure the flexibility of the prepared films [8]. Orodispersible films are also defined as dosage forms that can be given to the patient without the use of water because ODFs consist of polymers that allow them to be broken down rapidly by saliva and disintegrate in a few seconds, dissolving easily and being absorbed through the oral cavity or the tongue. After mucosal absorption, the drug enters the thin membranes of the oral cavity and is promptly bioavailable due to fast blood flow. The Food and Drug Administration (FDA) defined ODFs as dosage forms consisting of one or more APIs with an elastic nature so that, when placed on the tongue, they allow quick disintegration or dissolution by saliva.

The oral mucosa has greater power of penetration because of more blood vessels and the thin membrane structure—properties that provide good bioavailability. The higher the permeation, the higher the systemic bioavailability of drug contents, with avoidance of the first pass effect. Generally, ODFs are flexible and consist of a thin polymer layer, made with or without the use of plasticizers. They achieve patient compliance by lessening discomfort. In the literature, ODFs have been reported to resolve many issues associated with conventional dosage forms and improve initial drug effects, increase the durations of effects, minimize the frequency of doses, and aggregate the efficacy of drugs. By using this novel drug delivery system (film technology), the side effects of drugs are reduced, metabolism of drugs is increased, absorption is improved, and, most significantly, the bioavailability of drugs is increased. ODFs provide rapid dissolution, appropriate drug loading capacities, and improve the stability of drug formulations. Furthermore, they are non-toxic, biocompatible, and biodegradable [9,10,11,12].

Nowadays, ODFs are popular, having gained greater acceptance and are recognized in the pharmaceutical industry as convenient oral drug carriers. The existence of many polymers and production technologies have made it easy to develop a diverse range of ODFs. The European Medicines Agency (EMA) stated that ODFs are easily dissolved in the mucosal layer, from within a few seconds to a minute, after making contact with saliva and that they provide rapid bioavailability [10]. In 2010, the FDA approved the first ever prescription ODF named Zuplenz, which contains ondansetron as an API, and Suboxone was the second FDA-approved ODF. Since then, Listerine ODFs have become available on the market as breathe fresheners and have gained popularity worldwide. Currently, there are several ODFs available on the market, and doctors prescribe them. Some of the ODFs available on the market as prescription and OTC drugs are listed in Table 1. ODFs are manufactured in the form of a large sheet and cut according to the desired size and dosage [13]. ODFs have been developed for a wide range of drugs providing local action or systemic action. For local action, ODFs are used for toothaches, local anesthetic, cold sores, and oral ulcers [14]. For systemic action, they are used for treating cough, sore throat, migraine, gastric disorders, central nervous system disorder, pain, and nausea.

## 2. Merits and Demerits of ODFs

Orodispersible films are thin polymeric films which have a pleasant taste, are easily soluble after making contact with saliva, are easily ionized when they disintegrate on the tongue and penetrate the mucosal membrane, and provide prompt drug action. ODFs have many advantages over conventional dosage forms because they do not require any drinking of fluid for intake regardless of condition. They are stable, efficacious, have improved absorption, and higher bioavailability because of no first pass effect. ODFs have higher patient compliance because geriatric, paediatric, and paralyzed patients can easily take them without water and without choking problems. ODF technology helps to incorporate drugs at low doses as well as drugs that are incompatible with the gastrointestinal tract, and bioavailability problems are easily resolved. They provide quick effects in emergency conditions, such as asthmatic attack, migraine attack, angina attack, and in intraoral diseases. The development process of ODFs is easy and can be completed within a few days [10,21,22].

Orodispersible films have disadvantages because they require special equipment for packaging, and storage. This technology is not appropriate for drugs that are not ionized at oral pH or drugs that require large doses. ODFs are hygroscopic and therefore susceptible to deterioration, which makes them difficult to protect. They are easy to develop but have higher development costs than conventional dosage forms. Drugs that are absorbed by active diffusion cannot be incorporated into ODFs. They have fast dissolution and disintegration processes and so dose termination cannot be like that of tablets [10,21,22].

## 3. Classification of Orodispersible Films

ODFs are generally classified into three classes: type 1, according to dissolution; type 2, according to layering; and type 3, according to the nature of the API.

### 3.1. Type 1 ODFs

Type 1 ODFs are divided into three subclasses: fast, moderate, and slow. Films that dissolve within thirty seconds are termed fast-dissolving ODFs and have a thickness of around 50–150 μm; films that dissolve within one to thirty minutes are known as moderately dissolving ODFs; and slow-dissolving ODFs can take more than thirty minutes to dissolve. Fast-dissolving films are used in emergency conditions, while slow/moderately dissolving films are used to prepare nicotine-based products, as they help to lessen or eradicate cravings in patients who have used tobacco regularly and become dependent [12].

### 3.2. Type 2 ODFs

Type 2 ODFs are classified according to the number of layers they contain. Layers can be monolayers, bilayers, or multilayers. Monolayer oral films consist of an API, a film-forming polymer, and excipients, while bilayer or double layer films consist of one API layer and another taste-masking or permeation-enhancing layer. In multilayer films, the API layer is sandwiched between two layers.

### 3.3. Type 3 ODFs

Type 3 ODFs are further classified according to API source, which may be synthetic, e.g., sildenafil [23], or natural (animal or plant), e.g., ginger and turmeric [24,25]. Films prepared using minerals, vaccines, vitamins, or micronutrients constitute the other class of type 3 ODFs, e.g., vitamin D ODFs. All of these ODFs contain prescription drugs or over-the-counter drugs, while ODFs prepared from plant sources are difficult to fabricate [26]. Many companies have patented their ODFs and some examples of companies that have patented ODFs are listed in Table 2.

## 4. Formulation of Orodispersible Films

ODFs must have a pleasant taste, be of appropriate size, and be packed in suitable packing material. To ensure all these properties, drugs and excipients must have good stability, the required solubility and permeability, and the dose of the incorporated drug must be small dose with a low molecular weight (MW). A good ODF can be formulated using the desired active compound, either an active neutraceutical or a pharmaceutical ingredient, and excipients (film formers, stabilizers, plasticizers, sweeteners, and thickeners) [32].

### 4.1. Drugs

The most important quality of a drug candidate for use in an ODF is that it belong to BCS class 1, having high permeability and solubility, as well as a low MW with administrability at a low dose. Nowadays, however, companies and researchers are trying to prepare ODFs with drugs that belong to BCS classes 2 and 3. Researchers are also trying to prepare drugs with better tastes or with the masking of unpleasant tastes for better patient compliance. A list of drug candidates having low doses and low molecular sizes are presented in Table 3. The projected drug doses for ODF preparation generally range from 0.3 to 100 mg [12]. Other than pharmaceutical ingredients films, ODFs have also been prepared using plant leaf extracts that have therapeutic activities, e.g., cannabinoids. The preparation methods for ODFs seems to be easy but there are two difficult issues, namely, the unpleasant tastes of drugs and dose uniformity [33]. The taste of drugs is the main issue regarding the compliance of patients. In ODFs, drugs are incorporated directly into films but when absorbed on the tongue, after dissolution, bitter tastes can cause noncompliance in patients. To improve patient compliance, an obstruction method is used to mask or block the offensive taste. By obstructing the drug, the bitter taste is concealed. Some examples are obstruction of ketoprofen using cholestyramine resin, as well as cooling agents, taste-blocking agents, and sweetening agents. The bitter taste of sildenafil citrate was masked by using sodium bicarbonate and sodium hydroxide. Recently, researchers have also prepared ODFs of dimenhydrinate by masking with sulfobutylether-β-cyclodextrin and the taste masking was checked using an electronic taste-sensing system [34]. Taste masking can also be achieved by altering film composition (film-formers and drugs at a ratio of 9:1). Taste masking also helps to reduce drug load. However, by using obscuration methods, the taste masking of bitter drugs can be achieved easily. Another major problem in ODF formulation is drug agglomeration, which causes non-uniformity. Many companies have tried to resolve this issue in vain; to overcome it, they have made use of multilayer formulations. However, multilayer film formulation is expensive and time consuming [35].

### 4.2. Excipients

Out of all the excipients used in the formulation of ODFs, the film former plays an important role and is one of the key constituents. Maintaining the balance between disintegration times and the mechanical properties of ODFs, polymers, concentrations, and types is the main issue because the properties of polymers are affected by molecular weight [36,37]. Pullulan, polyvinyl alcohol (PVA), and cellulose derivatives are the commonest polymers that are used in the preparation of films [38,39,40]. Some of the examples of mixtures of polymers used in ODFs are methacrylic acid and hypromellose [41], croscarmellose and PVA [42], and macrogol-PVA and povidone [43]. A class of substances that help to provide flexibility in films are plasticizers, which aid the fabrication of films. In the literature, researchers have studied the impact of plasticizers on ODF preparation, and the selection of type and amount is an important consideration [44]. ODFs or rapidly disintegrating films are prepared with the aim of disintegrating or dissolving in the oral cavity when they are placed on the tongue. Some drugs have bitter tastes, creating a significant challenge in the development of this novel drug delivery system, and taste maskers can also be used as excipients in the preparation of ODFs. Polymers are useful in the development of ODFs and maintain tensile strength. Polymers from natural sources are mostly used in film formulations and are comprehensively cast in OTC films used for sore throat, cough, and breath fresheners. Gums (carrageenan, pectin, and agar) from plant sources can also be used in oral films [32,45]. Some of the excipients and their roles are enumerated in Table 4.

## 5. Techniques for Manufacturing ODFs

**Figure 1:**
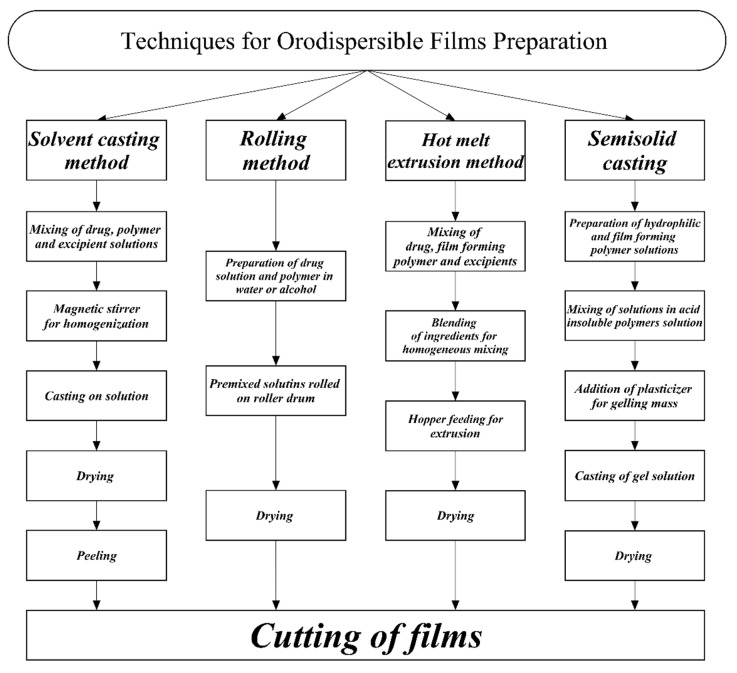
Flowchart of processes involved in the preparation of orodispersible films.

The manufacturing of ODFs is not an easy process and a number of techniques have been developed to manufacture them, such as the solvent casting method, the hot-melt extrusion method, the rolling method, semi-solid casting, and solid dispersion extrusion. Hot-melt extrusion and the solvent casting method are the commonest methods used by researchers and industries for the manufacture of films [54,55,56]. A flowchart of techniques and processes used in the preparation of orodispersible films is presented in Figure 1.

### 5.1. Solvent Casting Method

Solvent casting is one of the oldest methods used for the preparation of ODFs. It is an hydrous method, and thermostable and thermolabile drugs in dosage form are prepared via this method [57]. For the preparation of active pharmaceutical ingredients or plant extracts, first, active substances are dissolved in distilled water or any volatile solvent in which the drugs can dissolve easily and stirred well for uniformity with a magnetic stirrer. Solvents are selected according to the properties of the active ingredients. These properties include the compatibility of the active ingredient with excipients, especially film-forming polymers; solvent–drug compatibility; temperature sensitivity; and polymorphic nature. The film-forming polymer, colouring agent, plasticizer, and all the required excipients are separately prepared in distilled water. After preparing the required solution, the solution is again stirred for uniformity, and this material is called the film dope. The film dope is then spread on Petri dishes at laboratory scale, and the Petri dishes are placed in a hot oven at 40–50 °C for 24 h. After complete drying, the films are dispatched off and cut into the desired sizes and stored in aluminium foil for characterization. At industrial scale, the film dope is spread using the solvent cast film deposition methods on a roll of impregnated paper. The spread media is passed from a convection chamber to drive off the solvents. The films are cut into small pieces and wrapped separately in aluminium foil or are placed in air-tight pouches after drying. The packaging of films involves some precautions to avoid the effects of moisture. Moisture is one of the factors which disturbs the stability and mechanical properties of films. Moreover, the viscosity of solutions can only be maintained by controlling the temperature [58,59,60,61,62,63,64].

The solvent casting method is the best method for preparing heat- and light-sensitive active ingredients because lower temperatures are required by the volatile ingredients and for removing any solvent from the films. However, this method has some limitations, as during preparation, trace amounts of solvents can be left which hinder compendial compliance. Moreover, flammable solvents, such as methanol and ethanol, or volatile solvents require special protection to avoid fire [65].

### 5.2. Hot-Melt Extrusion Method

Oral thin films were formerly developed by the solvent casting method. While this approach to preparing films permits flexibility, clarity, uniformity, and the desired thicknesses to facilitate the loading of drugs, it is restricted by decreased elasticity and enlarged tensile strength [66]. One more drawback of films prepared via solvent casting methods is the use of organic solvents for a number of polymers that are insoluble in water. Organic solvents are hazardous and their residues cause complications in the disposal of waste material, creating many environmental issues [67,68]. To resolve these issues, an alternative method was required by pharma industries and the hot-melt extrusion method was found to have several advantages. Firstly, there is no need for any solvents for oral film processing. Moreover, with this method, extrudates can be obtained in a single step, which makes it cost-effective, as during processing there is no need to compress drugs and excipients. The melting of active substances and polymers into a liquid state followed by mixing permits a uniform distribution of particles that promotes the bioavailability of drugs [69].

The hot-melt extrusion method was used for the preparation of sustained release tablets and granules in transmucosal drug delivery systems and in transdermal drug delivery systems such as skin patches in the past [70]. This method was reported by a number of researchers for the manufacture of ODFs. However, over the last few decades this method has gained in popularity for preparing ODSs. In this technique, one or more drugs can easily be extruded to achieve the desired dosage forms for delivering drugs. With this method, drugs and excipients are easily modified into film form for drug delivery instead of using the solvent casting method [71]. In the hot-melt extruded method, films are created by the blending of the drug, film former, plasticizer, surfactants, and other required excipients in appropriate amounts for uniform mixing. After mixing, the extrude is fed into a hopper and conveyed to a heated barrel where homogeneous films are developed with thicknesses of less than 1 mm. Sometimes additives are also added in the initial processing to ensure the adherence of films at the mucosal surface [72].

In the future, the manufacture of films by the hot-melt extrusion method will provide opportunities to formulate films for gastroretentive drug delivery and multilayered films for transdermal drug release applications. In medical devices, drugs can be incorporated into catheters and biodegradable stents by hot-melt extrusion methods. These perspectives may increase innovation, research, and commercialization across research institutes, universities, pharmaceutical industries, and biotechnology industries.

### 5.3. Semi-Solid Casting

The semi-solid casting method is also used for the development of ODFs. In this method, water-soluble solutions and film forming polymers are prepared and then added to acid insoluble polymer solution. Plasticizers in the required ratios are added to the previously prepared solution with the aim of obtaining the required gel mass. Under controlled conditions, the prepared gel mass is cast in the form of films of 0.015–0.05-inch thickness [73].

### 5.4. Rolling Method

ODFs can also be prepared via the rolling method, in which drug solutions are rolled on a drum. The drug is dissolved in distilled water or a mixture of alcohol and water. After rolling the premixed solutions on the roller, the thin film is dried and cut into the desired sizes. The premixed solution comprises the active ingredient, polar solvent, film forming polymer, and the required excipients are added in the tank. The solution with the intended dose is fed by a controlled valve pump to obtain the desired thickness [74].

### 5.5. 3D Printing

Over the past few years, researchers have also tried to develop ODFs using a new technique, namely, 3D printing. This is an additive technique that relies on the deposition of different layers of ingredients [75]. Researchers have developed ODFs using 3D printing techniques, and in the final step of production the resultant is formed by the solidification of powder material or semi-solid material or by liquid materials. In 3D printing methods, extrusion technologies with fused deposition are the commonest means of developing drug delivery systems [76]. One example of this method is the fabrication of aripiprazole ODFs. Initially, aripiprazole filaments are prepared by hot-melt extrusion then mixed with PVA and moistened with ethanol before drying. The film filaments are prepared using an extruder. The blended powder is fed and extruded through the die at a constant speed. The film filament is then collected and further used to fabricate 3D-modeled ODFs. The fabricated ODFs have specific lengths, widths, and depths [77].

## 6. Characterization and Evaluation

The characterization and evaluation of prepared ODFs is important, and numerous methods, such as organoleptic, disintegration time, dissolution, surface pH visual inspection, moisture content, swelling index, mechanical properties, transparency, contact angle, and content uniformity tests, have been developed to assess prepared ODFs according to desired aims and objectives [78,79,80,81,82,83,84,85].

### 6.1. Organoleptic Evaluation

Organoleptic evaluation is an in vivo and in vitro taste evaluation method, and special, controlled human taste panels are used to conduct them. In in vivo evaluation, human volunteers are involved in the taste evaluation, and in vitro taste evaluation of films is performed using electronic taste sensing systems [34]. TS-5000Z (Insent) and α sstree (Alphamos) are commercially available taste sensing systems used for ODF evaluation. The TS-5000Z system is equipped with seven lipid membrane sensors corresponding to human taste attributes (three bitter taste sensors, and one each for salty, sour, umami and astringent taste). The other available system (α astree) is equipped with seven ChemFET-sensors for pharmaceutical use [86,87]. Taste sensing equipment has attracted interest in drug development, as unpleasant tastes reduce patient and therapy compliance in geriatric, paediatric, and bed-ridden patients. These electronic tongues provide a new approach to check films loaded with drugs having bad or poor tastes. In vitro taste sensing methods are suitable for checking the taste of dosage forms. Both in vitro and in vivo techniques are used to analyze the tastes of formulations and levels of sweetness in taste-masking formulation [88].

### 6.2. Surface Morphology

Surface morphology or visual inspection of ODFs gives evidence regarding transparency, homogeneity, and colour [81]. For this, light microscopy (LM) and scanning electron microscopy (SEM) have been used. SEM performance, due to advancement and uniform surfaces with an absence of pores, can be used to determine the high quality of ODFs. Films of 1 cm^2^ were placed on the stage of a microscope over a glass slide and the structure of film was observed at the micro-level, while SEM observed film structure more deeply [89].

### 6.3. Disintegration Time

In pharmacopoeias, numerous disintegration apparatuses are listed for use in the determination of film disintegration times (DTs). DT is a function of film composition because DT varies with formulations. Typically, ODFs disintegrate within 5–30 s. To date, no official pharmacopeial guidelines have been made available for determining the DTs of ODFs [79]. There are two methods for determining the DTs of ODF formulations by Petri dish method and slide frame methods, but mostly investigators use the Petri dish method [90].

#### 6.3.1. Slide Frame Method

In the slide frame method, films are clamped on a glass slide frame or a Petri dish. Drops of pure distilled water are poured onto the film and the time taken to dissolve is noted [85].

#### 6.3.2. Petri Dish Method

In this method, firstly place 10 mL of heated distilled water in the Petri dish and place 2 cm^2^ sized films in it. The Petri dish should then be shaken slightly to calculate the disintegration time of the film. The total time taken by the strip to break down is the disintegration time. For good results, the same process should be repeated thrice, with the results recorded as means and standard deviations [91].

### 6.4. In Vitro Dissolution Test

For in vitro dissolution testing, two official paddle and basket apparatuses have been used to determine film dissolution time. During the dissolution testing, sink conditions must be maintained. Occasionally, during dissolution, film floats over the medium and testing became difficult. This problem occurs mostly with a paddle apparatus; therefore, the basket apparatus method is preferred. In the context of a study, the media used in both apparatuses were phosphate-buffered solution of pH 6.8, 0.1 N HCL, purified water, gastric fluids, and intestinal fluids. Six aliquots of 5 mL were drawn after every minute and then at 8, 10, 12, 16, 20, and 30 min. The drawn samples were analyzed using a UV spectrophotometer [92].

### 6.5. Swelling Properties

Swelling properties of ODFs are determined because polymers are used for making films hydrophilic in nature [93]. The rate and degree of swelling in films play a pivotal role in the release of drugs. However, these properties are usually considered to check drug release patterns and the mucoadhesion of films [94]. Simulated saliva solution has been used to augment the swelling profile of films. The swelling of films was evaluated with the percentage of hydration. For this, firstly, films were weighed (W1) and immersed in simulated salivary fluid for a pre-set time. After that time, the samples were taken out, excess water was removed from the surfaces, and they were again weighed (W2). The percent hydration was calculated using the following formula [95,96]:

$$\mathrm{Hydration}\;\left(\%\right)=\frac{W2-W1}{W1}\times 100$$

To calculate the degree of swelling, the initial film weight (Wi) was noted and then the film was placed on a wire mesh and dipped into medium. The film weight was noted till no more increase in weight was observed after regular time intervals (Wf). The degree of swelling was calculated using the following formula [37]:

$$\mathrm{Degree}\;\mathrm{of}\;\mathrm{swelling}=\frac{\mathrm{Wf}-\mathrm{Wi}}{\mathrm{Wi}}$$

### 6.6. Surface pH

The surface pH of the buccal cavity is in the range of 5.5 to 7.4. ODFs are designed to quickly dissolve in the oral cavity after placement on the tongue, so ODF pH levels must be in the range of buccal cavity pH, as formulations having alkaline or very acidic pHs cause mucosal irritation. Randomly, films were selected for determining surface pH. The pH of dry film cannot be determined, so, first, film state was altered to measure pH by dissolving the films in 2 mL of distilled water. The electrode of the pH meter was placed on the surface of the solution and the reading was noted after 10 min to allow the stabilization of the pH value [97].

### 6.7. Moisture Content

Moisture content (MC) loss is an important consideration in determining a film’s hygroscopicity. In one study, this evaluation was determined by weighing four films which were subsequently put in a desiccator or hot air oven at 50 °C till a constant weight of films was achieved [98]. MC was calculated using the following equation:

$$\%\;\mathrm{Moisture}\;\mathrm{Content}=\frac{\mathrm{Wo}-\mathrm{Wd}}{\mathrm{Wo}}\times 100$$

where Wd is the dried weight and Wo is the initial weight of the film.

### 6.8. Mechanical Properties

Mechanical properties of ODFs were calculated on the basis of results obtained for surface thickness, percent elongation, Young’s modulus, tear resistance folding endurance, and tensile strength [99]. It was known from the literature that weak and soft polymers shows less elongation, less tensile strength, and lower Young’s modulus values as compared to tough and hard polymer [100]. Furthermore, manufacturing methods disturb the mechanical properties of films.

#### 6.8.1. Thickness

Film thickness is determined in order to know the uniformity of drug contents and is calculated using a micrometer. Homogeneity in film thickness is significant for ascertaining drug dose accuracy. The thicknesses of films were measured from the four corners and from the centres of films and mean values were recorded [101].

#### 6.8.2. Dryness

Dryness or tack tests are performed to determine the adherence of films with a piece of paper. Tack is the persistence of films observed when a piece of paper is pressed between films. Practically, there are different stages of drying films that are known as dry print-free, tack-free, dry-through, dust-free, set-to-touch, dry-hard, dry-to-recoat, and dry-to-touch. This test has been used to assess film dryness [78,79].

#### 6.8.3. Tensile Strength

The tensile strength (TS) test is an important mechanical test to observe the flexibility and strength of films. Tensile strength in the ODFs is defined as the maximum pressure and force that a film can bear before breakage. TS is calculated by dividing the force which causes breakage in a film by the initial size of the film [102].

#### 6.8.4. Percent Elongation

Elongation is a transformation in the size and shape of any object under stress conditions [18]. It helps to predict the ductility of polymers used in formulations and can be measured using a texture analyzer. The percent elongation specifies the stretching ability of a film before being broken down; however, percent elongation can easily be calculated as the difference in the initial length of film and the changed length after film breakage [103,104]. It can be calculated using the following mathematical expression [105]:

$$\%\;\mathrm{Elongation}=\frac{\mathrm{Change}\;\mathrm{in}\;\mathrm{length}\;\mathrm{at}\;\mathrm{break}}{\mathrm{Initial}\;\mathrm{length}\;}\times 100$$

#### 6.8.5. Young’s Modulus

Young’s modulus reflects the extent of film elasticity or stiffness. It specifies resistance to deformation films and can be determined by drawing a graph between the stress–strain curves and the slopes showing Young’s moduli. The higher the slope, the higher the tensile modulus, and vice versa [106]. Put simply, brittle and hard films exhibit higher Young’s modulus values. Results can be presented as the ratio of applied stress over strain that can be calculated by using the following formula [107]:

$$\mathrm{Young}’s\;\mathrm{modulus}=\frac{\mathrm{Slope}\times 100}{\mathrm{Film}\;\mathrm{thickness}\;\times \mathrm{Speed}}$$

#### 6.8.6. Folding Endurance

Folding endurance (FE) is an evaluation test used to assess the mechanical properties of ODFs. It is an important parameter with respect to delivering the dosage form accurately to the patient without breakage. The flexibility of films can easily be measured with FE. It is calculated by folding a film repeatedly at 180° angles until cracks appear. There is a direct relation between FE and the mechanical power of films. The higher the FE value, the higher will be the mechanical strength, and vice versa. However, plasticizer concentration indirectly affects the FE value because mechanical strength is influenced by the amount of plasticizer in the film formulation. For better results, tests are to be repeated thrice [108,109].

### 6.9. Content Uniformity

The content uniformity of formulations can be calculated using a standard assay method described in pharmacopoeias. It is estimated to check the drugs content present in films [79]. For this test, first dissolve the ODF of size 1 cm^2^ in 100 mL buffer solution. From that solution, take aliquots of 2 mL and dilute with buffer solution up to 10 mL. The diluted sample is then to be checked with a UV–Vis spectrophotometer by setting the absorbance according to the active ingredient used. The absorbance value helps to estimate the amount of drug in the film to check drug content uniformity [110].

## 7. Packaging of Films

Packaging of films is important to maintain stability during storage and retain the mechanical properties of ODF formulations. Packaging material acts as a barrier to provide protection from light, heat, oxygen, and moisture. Commercially available packaging materials for the storage of films include foil paper, aluminum pouches, plastic pouches, and blister packs. However, none of them is effective in providing stability in the storage of film products [85]. Aluminum foil packaging is the commonest and ideal packaging for films because it preserves the ODFs from light, heat, moisture, and degradation. Films not only need primary packaging; they are also stored in secondary packaging containers to achieve airtight environments [18]. Commercially, different sizes of ODFs and simple films are available on the market of size 1 × 2 cm^2^, 2 × 2 cm^2^, and 3 × 2 cm^2^ [110]. The packing of ODFs is economical, safe, and easy, though time-consuming; it also provides ease in handing, and films of any size and dimensions can be packed in pouches [111]. On the market, numerous films are available that are packed as single doses or multiple-unit doses. A single-dose film from Pfizer consumer healthcare is available on the market under the name Pocketpaks™ for breath freshening. Similarly, APR-Labtec launched a packaging system for multiple doses of Rapid^®^ films containing six films [110]. The multiple film packaging system is an automated and computer-driven process for ease of use and to increase patient compliance [18].

## 8. Conclusions and Future Perspective

The drug delivery system of ODFs has become popular over the past decade. Numerous drawbacks in terms of administration, bioavailability, solubility, and taste associated with conventional dosage forms decrease the compliance of patients, but the novel drug delivery system of oral thin films made from natural, synthetic, and semi-synthetic polymers has been used to promote compliance. Different techniques are available to develop films for oral routes to deliver drugs instantly; using such techniques we can also design films for delivering drugs via ocular, rectal, vaginal, and transdermal routes. Consequently, novel films can be used as alternatives to conventional dosage forms and can easily overcome the problems associated with conventional dosage forms. The novel dosage film formulation encounters some challenges during formulation and manufacture, but all these issues have been addressed by the optimization of formulations. The future of film technology looks promising for delivering drugs by any route to overcome the issues connected with conventional technologies.

## Figures and Tables

**Table 1:** ODFs available on the market as OTC and prescription drugs.

Brand Name	API	ODF Use	Category	Ref.
Chloraseptic Sore Throat Relief Strips	Benzocaine	For sore throat relief	OTC	[15]
Orajel Kids Sore Throat Relief Strips	Benzocaine	For children’s sore throat relief	OTC	[16]
Snoreeze Oral Strips	Peppermint oil, vitamin E	For snoring relief	OTC	[16]
Benadryl Allergy quick dissolve strip	Diphenhydramine HCL	For allergy	OTC	[16]
Gas-X Thin Strips	Simethicone	For reducing bloating	OTC	[10]
Pedia-Lax Quick Dissolve Strip	Sennosides	For treating constipation	OTC	[17]
Supress Cough Strips	Menthol	For cough	OTC	[18]
Risperidon hexal	Risperidone	For treating schizophrenia	Rx	[1]
Suboxone Sublingual Film	Buprenorphine, naloxone	For treating opioid use disorder	Rx	[16]
Sudafed PE	Phenylephrine HCL	For relief of stuffy nose	OTC	[19]
Theraflu Thin Strips multi symptom	Diphenhydramine HCL	For the common cold	OTC	[18]
Theraflu Thin Strips Long Acting Cough	Dextromethorphan HBR	For long-acting cough	OTC	[18]
Triaminic Thin Strips allergy	Diphenhydramine HCL	For allergy	OTC	[18]
Triaminic Thin Strips cold with stuffy nose	Phenylephrine HCL	For cold with a stuffy nose	OTC	[18]
Triaminic Thin Strips Day Time Cold & Cough	Dextromethorphan HBR, phenylephrine HCL	For day-time cold and cough	OTC	[16]
Triaminic Thin Strips Night Time Cold & Cough	Diphenhydramine, phenylephrine HCL	For night-time cold and cough	OTC	[18]
Zuplenz	Ondansetron	For nausea and vomiting	Rx	[16]
NiQuitin	Nicotine	For nicotine withdrawal symptoms	Rx	[19]
Zolmitriptan oral film	Zolmitriptan	For migraine	Rx	[19]
Sildenafil Sandoz Orodispersible Film	Sildenafil	For treating erectile dysfunction	Rx	[20]
IvyFilm	Hedera helix extract (http://ivyfilm.co.za/)	For relief of productive cough	OTC	[20]
Clobazam OSF	Clobazam	Used to treat seizures	Rx	[20]
Zuplenz	Ondansetron	For nausea and vomiting	Rx	[19]

**Table 2:** List of companies that have patented ODFs.

Patent Number	Company	Layers	Ref.
20,010,022,964	Johnson and Johnson Consumer Inc.	Monolayer	[27]
5,047,244	Actavis Laboratories	Bilayer	[28]
8,865,202	LTS Lohmann Therapie-Systeme GmbH and Co. KG	Monolayer	[29]
4,517,173	Nippon Soda Co. Ltd.	Bilayer	[30]
4,876,092	Teikoku Seiyaku Co. Ltd.	Bilayer	[31]

**Table 3:** Potential drugs for formulation in ODFs, along with their doses.

Drug	Drug Class	BCS Class	Dose (mg)	Category
Chlorpheniramine	Antihistamine	1	4–12	OTC
Loratadine	Antihistamine	2	5–10	OTC
Phenylephrine	Antihistamine		5–10	OTC
Diphenhydramine	Antihistamine	1	12.5–60	Rx
Dextromethorphan	Antitussives	2	10–30	OTC
Sildenafil	PDE inhibitors	1	25–100	Rx
Ketoprofen	NSAID	2	12.5–25	OTC
Sumatriptan	SSRA	3	35–70	Rx
Zolmitriptan	SSRA	3	2.5	Rx
Loperamide	Antidiarrheal	2	2	OTC
Famotidine	H2 blockers	3	5–10	Rx
Nicotine	NCA	1	1–15	Rx
Pseudoephedrine	Nasal decongestants	1	15–60	OTC
Atorvastatin	HMG-CoA RI	2	5–80	Rx
Valdecoxib	Cox-2 inhibitor	2	5–20	Rx
Amlodipine	CCB	1	2.5–10	Rx
Rofecoxib	NSAID	2	5–25	Rx
Setraline	SSRI	2	10–100	Rx
Ziprasidone	Antipsychotics	2	20–80	Rx
Eletriptan	SSRI	1	10–40	Rx
Nitroglycerin	Vasodilators		0.3–0.6	Rx

BCS, Biopharmaceutical classification system; OTC, Over the counter; Rx, Prescription; PDE, Phosphodiesterase; NSAID, Non-steroidal anti-inflammatory drug; SSRI, Selective serotonin receptor agonists; NCA, *Nicotinic* cholinergic agonist; HMG-CoA RI, HMG-CoA reductase inhibitor; H2, Histamine-2; COX-2, Cyclooxygenase-2 inhibitor; CCB, Calcium channel blocker.

**Table 4:** Excipient and its role in ODFs formulation.

Excipient	Conc.	Role of Excipient	Example of Excipient
Film-forming polymers	40–50%	They provide shape, elasticity, fast disintegration, and mechanical strength in films	Sodium carboxy methyl cellulose, hydroxyl ethyl cellulose, hydroxyl propyl methyl cellulose, pectin, pullulan, gelatin, sodium alginate, starch, maltodextrin, methacrylic acid, xanthan gum, guar gum, locust bean gum, carrageenan, chitosan, polyvinyl pyrrolidone, polyvinyl alcohol, polyethylene oxide, polyvinyl acetate, polyvinyl pyrrolidone
Plasticizers	0–20%	They provide elongation, tensile strength, and plasticity; improve absorption and solubility; prevent crushing; and reduce brittleness and glass transition temperature	Mannitol, glycerol, sorbitol, citric acid macrogol, propylene glycol, polyethylene glycols, phthalate derivatives (dibutyl, diethyl, dimethyl), citrate derivatives (triacetin, acetyl citrate, triethyl, tributyl)
Sweetening agents	0–10%	They are used to improve the taste of films for patient compliance	Glucose, fructose, sucrose, sucralose, maltose, sorbitol, mannitol, stevioside sodium, ribose, cyclamate salts, aspartame, thaumatin, xylose, ribose, flavored essences, cyclamate, oleoresins
Saliva stimulants	0–10%	They increase saliva production	Ascorbic acid, citric acid, tartaric acid, malic acid, lactic acid
Taste maskers	0–10%	They are used to mask nauseating and bitter tastes for patient compliance	Hydroxypropyl-β-cyclodextrin, maltodextrin, sulfobutylether-β-cyclodextrin
Surfactants	0–10%	They help to disintegrate films in seconds and allow dispersion and solubilization	Poloxamer, sodium lauryl sulfate, polysorbate, laureth-a, sucrose esters, dodecyl maltoside, cetyl trimethylammonium bromide
Refs. [1,3,16,34,36,37,46,47,48,49,50,51,52,53]			

## Data Availability

Not applicable.

## References

[1] Siddiqui M., Garg G., Sharma P.K. (2011). A short review on “A novel approach in oral fast dissolving drug delivery system and their patents”. Adv. Biol. Res..

[2] Chan R. (2016). Oral thin films–realms of possibility. ONdrugDelivery Mag..

[3] Patel A.R., Prajapati D.S., Raval J.A. (2010). Fast dissolving films (FDFs) as a newer venture in fast dissolving dosage forms. Int. J. Drug Dev. Res..

[4] Zaman M., Hassan R., Amjad M.W., Khan S.M., Raja M.A.G., Shah S.S., Siddique W., Aman W., Abid Z., Butt M.H. (2021). Formulation of instant disintegrating buccal films without using disintegrant: An in-vitro study. Pak. J. Pharm. Sci..

[5] Wright D., Tomlin S. (2011). CPD-How to help if a patient can’t swallow. Pharm. J..

[6] Bandari S., Mittapalli R.K., Gannu R. (2014). Orodispersible tablets: An overview. Asian J. Pharm..

[7] Mahboob M.B.H., Riaz T., Jamshaid M., Bashir I., Zulfiqar S. (2016). Oral films: A comprehensive review. Int. Curr. Pharm. J..

[8] Committees of the European Parliament (2016). European Pharmacopoeia Ninth Edition (PhEur 9.0).

[9] Hussain W., Kushwaha P., Rahman A., Akhtar J. (2017). Development and evaluation of fast dissolving film for oro-buccal drug delivery of chlorpromazine. Indian J. Pharm. Educ. Res..

[10] Karki S., Kim H., Na S.-J., Shin D., Jo K., Lee J. (2016). Thin films as an emerging platform for drug delivery. Asian J. Pharm. Sci..

[11] Özakar R.S., Özakar E. (2020). A Current Overview of Oral Thin Films. Oral İnce Filmlere Güncel Bir Bakış. Turk. J. Pharm. Sci..

[12] Gupta M.S., Kumar T.P., Gowda D.V. (2020). Orodispersible Thin Films: A New Patient Centered Innovation. J. Drug Deliv. Sci. Technol..

[13] Desai P.P., Date A.A., Patravale V.B. (2012). Overcoming poor oral bioavailability using nanoparticle formulations–opportunities and limitations. Drug Discov. Today Technol..

[14] Kumar V.D., Sharma I., Sharma V. (2011). A comprehensive review on fast dissolving tablet technology. J. Appl. Pharm. Sci..

[15] Mishra R., Amin A. (2011). Formulation and characterization of rapidly dissolving films of cetirizine hydrochloride using pullulan as a film forming agent. Indian J. Pharm. Educ. Res..

[16] Hoffmann E.M., Breitenbach A., Breitkreutz J. (2011). Advances in orodispersible films for drug delivery. Expert Opin. Drug Deliv..

[17] Krause J., Breitkreutz J. (2008). Improving drug delivery in paediatric medicine. Pharm. Med..

[18] Dixit R.P., Puthli S.P. (2009). Oral strip technology: Overview and future potential. J. Control. Release.

[19] Preis M.K. (2014). Oromucosal Film Preparations for Pharmaceutical Use-Formulation Development and Analytical Characterization.

[20] Wasilewska K., Winnicka K. (2019). How to assess orodispersible film quality? A review of applied methods and their modifications. Acta Pharm..

[21] Kathpalia H., Gupte A. (2013). An introduction to fast dissolving oral thin film drug delivery systems: A review. Curr. Drug Deliv..

[22] Saini P., Kumar A., Sharma P., Visht S. (2012). Fast disintegrating oral films: A recent trend of drug delivery. Int. J. Drug Dev. Res..

[23] Radicioni M., Castiglioni C., Giori A., Cupone I., Frangione V., Rovati S. (2017). Bioequivalence study of a new sildenafil 100 mg orodispersible film compared to the conventional film-coated 100 mg tablet administered to healthy male volunteers. Drug Des. Dev. Ther..

[24] Gopi S., Amalraj A., Kalarikkal N., Zhang J., Thomas S., Guo Q. (2019). Preparation and characterization of nanocomposite films based on gum arabic, maltodextrin and polyethylene glycol reinforced with turmeric nanofiber isolated from turmeric spent. Mater. Sci. Eng. C.

[25] Daud A., Bonde M., Sapkal N. (2014). Development of Zingiber officinale in oral dissolving films: Effect of polymers on in vitro, in vivo parameters and clinical efficacy. Asian J. Pharm..

[26] Gupta M.S., Kumar T.P. (2020). The potential of ODFs as carriers for drugs/vaccines against COVID-19. Drug Dev. Ind. Pharm..

[27] Leung S.-H.S., Leone R.S., Kumar L.D., Kulkarni N., Sorg A.F. (2001). Fast Dissolving Orally Consumable Films. Google Patent.

[28] Sanvordeker D.R., Leung S.-H.S. (1991). Mucoadhesive Carrier for Delivery of Therapeutical Agent. Google Patent.

[29] Zerbe H.G., Guo J.-H., Serino A. (2014). Water Soluble Film for Oral Administration with Instant Wettability. Google Patent.

[30] Kizawa H., Fujiyama N., Kobayashi J., Ito A. (1985). Mucous Membrane-Adhering Film Preparation and Process for Its Preparation. Google Patent.

[31] Mizobuchi T., Ohji A., Sakoh S., Muguruma Y. (1988). Sheet-Shaped Adhesive Preparation Applicable to Oral Cavity. Google Patent.

[32] Siddique W., Sarfraz R., Zaman M., Butt M., Hayat Z., Gul M., Gul M., Asghar F. (2021). Impact of polymer and plasticizer on mechanical properties of film: A quality by design approach. Lat. Am. J. Pharm..

[33] Allen E., Davidson R., LaRosa T., Reid D. (2019). Oral Dissolvable Film That Includes Plant Extract. Google Patent.

[34] Preis M., Pein M., Breitkreutz J. (2012). Development of a taste-masked orodispersible film containing dimenhydrinate. Pharmaceutics.

[35] Horstmann M., Laux W., Hungerbach S. (1997). Rapidly Disintegrating Sheet-Like Presentations of Multiple Dosage Units. Google Patent.

[36] Garsuch V., Breitkreutz J. (2010). Comparative investigations on different polymers for the preparation of fast-dissolving oral films. J. Pharm. Pharmacol..

[37] Irfan M., Rabel S., Bukhtar Q., Qadir M.I., Jabeen F., Khan A. (2016). Orally disintegrating films: A modern expansion in drug delivery system. Saudi Pharm. J..

[38] Boateng J.S., Matthews K.H., Auffret A.D., Humphrey M.J., Stevens H.N., Eccleston G.M. (2009). In vitro drug release studies of polymeric freeze-dried wafers and solvent-cast films using paracetamol as a model soluble drug. Int. J. Pharm..

[39] Boateng J.S., Stevens H.N., Eccleston G.M., Auffret A.D., Humphrey M.J., Matthews K.H. (2009). Development and mechanical characterization of solvent-cast polymeric films as potential drug delivery systems to mucosal surfaces. Drug Dev. Ind. Pharm..

[40] Pathare Y.S., Hastak V.S., Bajaj A.N. (2013). Polymers used for fast disintegrating oral films: A review. Polymer.

[41] Sharma R., Parikh R., Gohel M., Soniwala M. (2007). Development of taste masked film of valdecoxib for oral use. Indian J. Pharm. Sci..

[42] Kulkarni A., Deokule H., Mane M., Ghadge D. (2010). Exploration of different polymers for use in the formulation of oral fast dissolving strips. J. Curr. Pharm. Res..

[43] Ali S., Quadir A. (2007). High molecular weight povidone polymer-based films for fast dissolving drug delivery applications. Drug Deliv. Technol..

[44] Cao N., Yang X., Fu Y. (2009). Effects of various plasticizers on mechanical and water vapor barrier properties of gelatin films. Food Hydrocoll..

[45] Hanif M., Zaman M., Chaurasiya V. (2015). Polymers used in buccal film: A review. Des. Monomers Polym..

[46] Giovino C., Ayensu I., Tetteh J., Boateng J.S. (2013). An integrated buccal delivery system combining chitosan films impregnated with peptide loaded PEG-b-PLA nanoparticles. Colloids Surf. B Biointerfaces.

[47] Murata Y., Isobe T., Kofuji K., Nishida N., Kamaguchi R. (2010). Preparation of fast dissolving films for oral dosage from natural polysaccharides. Materials.

[48] Nagar P., Chauhan I., Yasir M. (2011). Insights into polymers: Film formers in mouth dissolving films. Drug Invent. Today.

[49] Arora L., Chakraborty T. (2017). A review on new generation orodispersible films and its novel approaches. Indo Am. J. Pharm. Res..

[50] Shinkar D.M., Dhake A.S., Setty C.M. (2012). Drug delivery from the oral cavity: A focus on mucoadhesive. PDA J. Pharm. Sci. Technol..

[51] Hanif M., Zaman M. (2017). Thiolation of arabinoxylan and its application in the fabrication of controlled release mucoadhesive oral films. DARU J. Pharm. Sci..

[52] Zaman M., Hanif M., Shaheryar Z.A. (2018). Development of Tizanidine HCl-Meloxicam loaded mucoadhesive buccal films: In-vitro and in-vivo evaluation. PLoS ONE.

[53] Zaman M., Hanif M., Qaiser A.A. (2016). Effect of polymer and plasticizer on thin polymeric buccal films of meloxicam designed by using central composite rotatable design. Acta Pol. Pharm..

[54] Mishra R., Amin A. (2011). Manufacturing techniques of orally dissolving films. Pharm. Technol..

[55] Zaman M., Hanif M., Sultana K. (2018). Synthesis of thiolated arabinoxylan and its application as sustained release mucoadhesive film former. Biomed. Mater..

[56] Zaman M., Hanif M., Khan M.A. (2018). Arabinoxylan-based mucoadhesive oral films of tizanidine HCL designed and optimized using central composite rotatable design. Polym.-Plast. Technol. Eng..

[57] Zaman M., Hassan R., Razzaq S., Mahmood A., Amjad M.W., Raja M.A.G., Qaisar A.A., Majeed A., Hanif M., Tahir R.A. (2020). Fabrication of polyvinyl alcohol based fast dissolving oral strips of sumatriptan succinate and metoclopramide HCL. Sci. Prog..

[58] Kathpalia H., Patil A. (2017). Formulation and evaluation of orally disintegrating films of levocetirizine dihydrochloride. Indian J. Pharm. Sci..

[59] Reza K.H., Chakraborty P. (2016). Recent industrial development in oral thin film technology: An overview. PharmaTutor.

[60] Schruben D.L., Gonzalez P. (2000). Dispersity improvement in solvent casting particle/polymer composite. Polym. Eng. Sci..

[61] Verma S. (2018). A review on conventional and modern techniques to develop orodispersible films. Asian J. Pharm..

[62] Tsujimoto T. (2008). Solvent Casting Process. Google Patent.

[63] Nagaraju T., Gowthami R., Rajashekar M., Sandeep S., Mallesham M., Sathish D., Kumar Y.S. (2013). Comprehensive review on oral disintegrating films. Curr. Drug Deliv..

[64] Zaman M., Hanif M. (2018). In vitro and ex vivo assessment of hydrophilic polymer-and plasticizer-based thin buccal films designed by using central composite rotatable design for the delivery of meloxicam. Adv. Polym. Technol..

[65] Melegari C. (2016). Study of Different Technologies for Film Coating of Drug Layered Pellets Using Ethylcellulose as Functional Polymer.

[66] Maniruzzaman M., Boateng J.S., Snowden M.J., Douroumis D. (2012). A review of hot-melt extrusion: Process technology to pharmaceutical products. Int. Sch. Res. Not..

[67] Barnhart S.D. (2008). Thin Film Oral Dosage Forms. Modified-Release Drug Delivery Technology.

[68] Palem C.R., Battu S.K., Maddineni S., Gannu R., Repka M.A., Yamsani M.R. (2013). Oral transmucosal delivery of domperidone from immediate release films produced via hot-melt extrusion technology. Pharm. Dev. Technol..

[69] McGinity J.W., Koleng J., Repka M., Zhang F. (2007). Hot-melt extrusion technology. Encycl. Pharm. Technol..

[70] Patil H., Tiwari R.V., Repka M.A. (2016). Hot-melt extrusion: From theory to application in pharmaceutical formulation. AAPS PharmSciTech.

[71] Simões M.F., Pinto R.M., Simões S. (2019). Hot-melt extrusion in the pharmaceutical industry: Toward filing a new drug application. Drug Discov. Today.

[72] Tumuluri V.S., Kemper M.S., Lewis I.R., Prodduturi S., Majumdar S., Avery B.A., Repka M.A. (2008). Off-line and on-line measurements of drug-loaded hot-melt extruded films using Raman spectroscopy. Int. J. Pharm..

[73] Arya A., Chandra A., Sharma V., Pathak K. (2010). Fast dissolving oral films: An innovative drug delivery system and dosage form. Int. J. ChemTech Res..

[74] Yang R.K., Fuisz R.C., Myers G.L., Fuisz J.M. (2008). Thin Film with Non-Self-Aggregating Uniform Heterogeneity and Drug Delivery Systems Made Therefrom. Google Patent.

[75] Jamróz W., Szafraniec J., Kurek M., Jachowicz R. (2018). 3D printing in pharmaceutical and medical applications–recent achievements and challenges. Pharm. Res..

[76] Annaji M., Ramesh S., Poudel I., Govindarajulu M., Arnold R.D., Dhanasekaran M., Babu R.J. (2020). Application of extrusion-based 3D printed dosage forms in the treatment of chronic diseases. J. Pharm. Sci..

[77] Łyszczarz E., Brniak W., Szafraniec-Szczęsny J., Majka T.M., Majda D., Zych M., Pielichowski K., Jachowicz R. (2021). The impact of the preparation method on the properties of orodispersible films with aripiprazole: Electrospinning vs. casting and 3D printing methods. Pharmaceutics.

[78] Chaudhary H., Gauri S., Rathee P., Kumar V. (2013). Development and optimization of fast dissolving oro-dispersible films of granisetron HCl using Box–Behnken statistical design. Bull. Fac. Pharm. Cairo Univ..

[79] Bhyan B., Jangra S., Kaur M., Singh H. (2011). Orally fast dissolving films: Innovations in formulation and technology. Int. J. Pharm. Sci. Rev. Res..

[80] Bai G., Armenante P.M., Plank R.V., Gentzler M., Ford K., Harmon P. (2007). Hydrodynamic investigation of USP dissolution test apparatus II. J. Pharm. Sci..

[81] Raju S., Reddy P.S., Kumar V.A., Deepthi A., Reddy K.S., Reddy P.M. (2011). Flash release oral films of metoclopramide hydrochloride for pediatric use: Formulation and in-vitro evaluation. J. Chem. Pharm. Res..

[82] Patel R.S., Poddar S. (2009). Development and characterization of mucoadhesive buccal patches of salbutamol sulphate. Curr. Drug Deliv..

[83] Yellanki S., Jagtap S., Masareddy R. (2011). Dissofilm: A novel approach for delivery of phenobarbital; design and characterization. J. Young Pharm..

[84] Gorle A.P., Gattani S.G. (2009). Design and evaluation of polymeric ocular drug delivery system. Chem. Pharm. Bull..

[85] Patil P.C., Shrivastava S., Vaidehi S., Ashwini P. (2014). Oral fast dissolving drug delivery system: A modern approach for patient compliance. Int. J. Drug Regul. Aff..

[86] Siqueira W.L., Nicolau J. (2002). Stimulated whole saliva components in children with Down syndrome. Spec. Care Dent..

[87] Pein M., Eckert C., Preis M., Breitkreutz J. Taste Sensing System αAstree as Analytical Tool—Performance Qualification Using Caffeine Citrate as Model Substance. Proceedings of the 8th Pharmaceutics & Biopharmaceutics World Meeting.

[88] Zaman M., Hanif M., Amjad M.W., Mahmood A., Shah S., Raja M.A.G., Rasool S., Sarfraz R.M. (2019). Development of thiomer based buccal films for the enhancement of bioavailability: An in-vivo analysis. Pak. J. Pharm. Sci..

[89] Zaman M. (2018). Formulation and Evaluation of Tizanidine-Meloxicam Mucoadhesive Buccal Films by Central Composite Rotatable Design and Their Pharmacokinetic Studies.

[90] Zaman M., Murtaza H. (2018). Development and validation of RP-HPLC method for simultaneous estimation of tizanidine HCl and meloxicam in bilayer mucoadhesive buccal films. Acta Pol. Pharm. Drug Res..

[91] El-Setouhy D.A., Abd El-Malak N.S. (2010). Formulation of a novel tianeptine sodium orodispersible film. AAPS PharmSciTech.

[92] Anwar S., Zaman M., Raja M.A.G., Mahmood A., Amjad M.W. (2020). Rosuvastatin, Perindopril and Ezetimibe loaded instant release buccal films: Development and in vitro characterization. J. Appl. Biomed..

[93] Peh K.K., Wong C.F. (1999). Polymeric films as vehicle for buccal delivery: Swelling, mechanical, and bioadhesive properties. J. Pharm. Pharm. Sci..

[94] Aburahma M.H., Mahmoud A.A. (2011). Biodegradable ocular inserts for sustained delivery of brimonidine tartarate: Preparation and in vitro/in vivo evaluation. AAPS PharmSciTech.

[95] Baranowski P., Karolewicz B., Gajda M., Pluta J. (2014). Ophthalmic drug dosage forms: Characterisation and research methods. Sci. World J..

[96] Eroğlu H., Sargon M.F., Öner L. (2007). Chitosan formulations for steroid delivery: Effect of formulation variables on in vitro characteristics. Drug Dev. Ind. Pharm..

[97] Kunte S., Tandale P. (2010). Fast dissolving strips: A novel approach for the delivery of verapamil. J. Pharm. Bioallied Sci..

[98] Ahmad A., Butt M.H., Misbah S., Saleem R.T., Jamshaid M., Alvi M.N. (2020). Development and evaluation of orodispersible films by solvent casting method using eletriptan hydrobromide as a model drug. Lat. Am. J. Pharm..

[99] Preis M., Knop K., Breitkreutz J. (2014). Mechanical strength test for orodispersible and buccal films. Int. J. Pharm..

[100] Morales J.O., McConville J.T. (2011). Manufacture and characterization of mucoadhesive buccal films. Eur. J. Pharm. Biopharm..

[101] Bhupinder B., Sarita J. (2012). Formulation and evaluation of fast dissolving sublingual films of Rizatriptan Benzoate. Int. J. Drug Dev. Res..

[102] Khalil Y.I. (2015). Preparation and characterization of montelukast sodium (SMLT) as a dual sustained release buccal strips. Iraqi J. Pharm. Sci..

[103] Bonsu M.A., Ofori-Kwakye K., Kipo S.L., Boakye-Gyasi M.E., Fosu M.-A. (2016). Development of oral dissolvable films of diclofenac sodium for osteoarthritis using Albizia and Khaya gums as hydrophilic film formers. J. Drug Deliv..

[104] Alghamdi H., Svirskis D., Bunt C.R., Swift S., Rupenthal I.D. (2016). Azithromycin and Dexamethasone Loaded β-Glucan Films for the Treatment of Blepharitis. Drug Deliv. Lett..

[105] Koland M., Sandeep V., Charyulu N. (2010). Fast dissolving sublingual films of ondansetron hydrochloride: Effect of additives on in vitro drug release and mucosal permeation. J. Young Pharm..

[106] Wuzhu Y., Shifeng W., Jun L., Zhufeng Y. (2009). Determination of reduced Young’s modulus of thin films using indentation test. Acta Metall. Sin..

[107] Dinge A., Nagarsenker M. (2008). Formulation and evaluation of fast dissolving films for delivery of triclosan to the oral cavity. AAPS PharmSciTech.

[108] Prabhu P., Malli R., Koland M., Vijaynarayana K., D’Souza U., Harish N., Shastry C., Charyulu R. (2011). Formulation and evaluation of fast dissolving films of levocitirizine di hydrochloride. Int. J. Pharm. Investig..

[109] Gavaskar B., Kumar S.V., Sharan G., Rao Y.M. (2010). Overview on fast dissolving films. Int. J. Pharm. Pharm. Sci..

[110] Amin P.M., Gangurde A., Alai P. (2015). Oral film technology: Challenges and future scope for pharmaceutical industry. Int. J. Pharm. Pharm. Res..

[111] Sharma D., Kaur D., Verma S., Singh D., Singh M., Singh G., Garg R. (2015). Fast dissolving oral films technology: A recent trend for an innovative oral drug delivery system. Int. J. Drug Deliv..

